# [μ-1,2-Bis(diphenyl­phosphan­yl)benzene-κ^2^ *P*:*P*′]bis­[chloridogold(I)]

**DOI:** 10.1107/S1600536810052803

**Published:** 2010-12-24

**Authors:** Nobuto Yoshinari, Naoki Kitani, Toshiaki Tsukuda, Takumi Konno

**Affiliations:** a Department of Chemistry, Graduate School of Science, Osaka University, Toyonaka, Osaka 560-0043, Japan

## Abstract

In the crystal structure of the non-solvate form of the title compound, [Au_2_Cl_2_(C_30_H_24_P_2_)], two almost linear P—Au^I^—Cl units [175.87 (3) and 171.48 (3)°] are in a skewed arrangement with a Cl—Au⋯Au—Cl torsion angle of −65.29 (3)° so as to form an intra­molecular Au⋯Au inter­action [3.0563 (2) Å]. The complex mol­ecules are connected each other through inter­molecular C—H⋯π inter­actions, giving a sheet structure parallel to the *bc* plane.

## Related literature

For the crystal structure of the diethyl­ether solvate form of the title compound, [(AuCl)_2_(C_30_H_24_P_2_)]·(C_2_H_5_)_2_O, see: Mohamed *et al.* (2003 ▶). For closely related structures, see: Hashimoto *et al.* (2010 ▶).
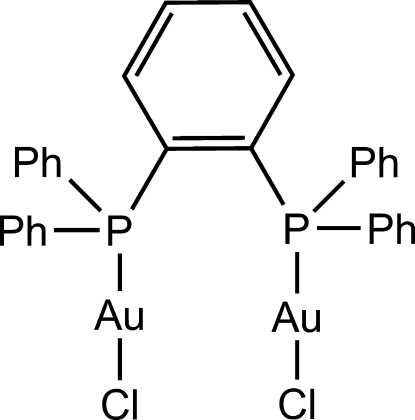


## Experimental

### 

#### Crystal data

[Au_2_Cl_2_(C_30_H_24_P_2_)]
*M* *_r_* = 911.27
Monoclinic, 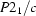

*a* = 13.0733 (2) Å
*b* = 12.4206 (2) Å
*c* = 17.4630 (3) Å
β = 96.795 (7)°
*V* = 2815.69 (8) Å^3^
*Z* = 4
Mo *K*α radiation
μ = 10.73 mm^−1^
*T* = 200 K
0.15 × 0.10 × 0.10 mm

#### Data collection

Rigaku R-AXIS VII diffractometer
Absorption correction: multi-scan (*ABSCOR*; Higashi, 1995 ▶) *T*_min_ = 0.189, *T*_max_ = 0.341
31710 measured reflections
6438 independent reflections
5897 reflections with *I* > 2σ(*I*)
*R*_int_ = 0.030

#### Refinement

*R*[*F* ^2^ > 2σ(*F* ^2^)] = 0.023
*wR*(*F* ^2^) = 0.041
*S* = 1.16
6438 reflections
325 parameters
H-atom parameters constrained
Δρ_max_ = 0.54 e Å^−3^
Δρ_min_ = −0.80 e Å^−3^

### 

Data collection: *PROCESS-AUTO* (Rigaku, 1998 ▶); cell refinement: *PROCESS-AUTO*; data reduction: *Yadokari-XG 2009* (Kabuto *et al.*, 2009 ▶); program(s) used to solve structure: *SHELXS97* (Sheldrick, 2008 ▶); program(s) used to refine structure: *SHELXL97* (Sheldrick, 2008 ▶); molecular graphics: *Mercury* (Macrae *et al.*, 2006 ▶) and *ORTEP-3 for Windows* (Farrugia, 1997 ▶); software used to prepare material for publication: *Yadokari-XG 2009* and *publCIF* (Westrip, 2010 ▶).

## Supplementary Material

Crystal structure: contains datablocks I, global. DOI: 10.1107/S1600536810052803/is2645sup1.cif

Structure factors: contains datablocks I. DOI: 10.1107/S1600536810052803/is2645Isup2.hkl

Additional supplementary materials:  crystallographic information; 3D view; checkCIF report

## Figures and Tables

**Table 1:** Selected bond lengths (Å)

Au1—P1	2.2256 (8)
Au1—Cl1	2.2739 (8)
Au2—P2	2.2279 (7)
Au2—Cl2	2.2792 (8)

**Table 2:** Hydrogen-bond geometry (Å, °) *Cg* is the centroid of the C25–C30 ring.

*D*—H⋯*A*	*D*—H	H⋯*A*	*D*⋯*A*	*D*—H⋯*A*
C15—H15⋯*Cg*^i^	0.95	2.82	3.569 (4)	137
C21—H21⋯*Cg*^ii^	0.95	2.84	3.559 (4)	134

Symmetry codes: (i) 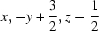; (ii) 
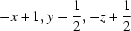
.

**Fig. 1.**
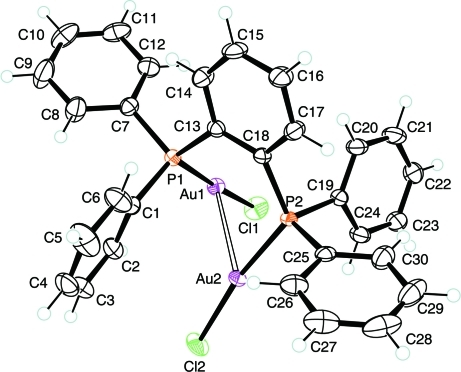
A view of molecular structure of the title compound, showing the atom-numbering scheme and 50% probability displacement ellipsoids.

**Fig. 2.**
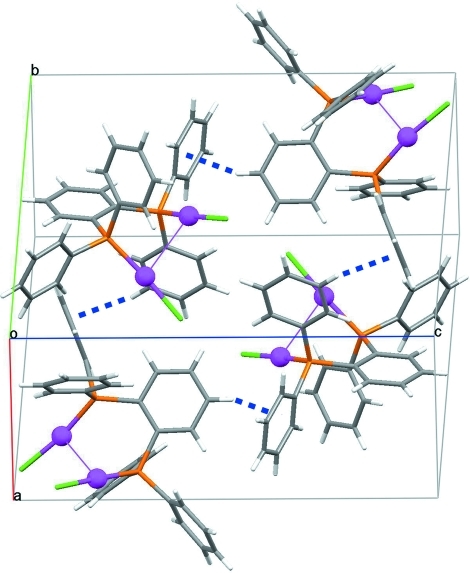
A crystal packing drawing of the title compound. The blue lines indicate C—H···π interactions.

## supplementary crystallographic information

### Comment

[(AuCl)_2_(diphosphine)]-type digold(I) complexes have been known as a good starting material to produce [(AuL)_2_(diphosphine)]-type digold(I) metallounits. Recently, we found that a digold(I) complex, [{Au(D-Hpen)}_2_(dppm)] (D-pen = D-penicillaminate, dppm = 1,2-bis(diphenylphosphino)methane), which was prepared from [(AuCl)_2_(dppm)] and D-pen, can act as a hexadentate-S_2_N_2_O_2_ metalloligand toward a Ni^II^ center to give a unique trinuclear Ni^II^Au^I^_2_ complex with a nine-membered metalloring, [NiAu_2_(D-pen)_2_(dppm)] (Hashimoto *et al.*, 2010). In the course of our study on a digold(I) metalloligand system having both D-pen and diphosphines, we started to use [(AuCl)_2_(dppbz)] (dppbz = *o*-phenylenebis(diphenylphosphine)) instead of [(AuCl)_2_(dppm)]. Herein, we report the crystal structure of the non-solvate form of [(AuCl)_2_(dppbz)] (I). The crystal structure of the diethylether solvate form of the title compound, [(AuCl)_2_(dppbz)].Et_2_O (II), has been reported by Mohamed *et al.* (2003).

The asymmetric unit of (I) contains only a complex molecule without a significant solvent accessible space, which is distinct from the solvated structure of (II) (Mohamed *et al.*, 2003). The complex molecule is composed of two [Au^I^Cl] units that are linked by a dppbz ligand through Au—P bonds, forming a digold(I) structure in [(AuCl)_2_(dppbz)] (Fig. 1). In (I), two approximately linear P—Au^I^—Cl units are skewed each other so as to form an intramolecular Au···Au interaction. This conformational feature is the same as that in (II). In the crystal (I), the Au···Au distance [3.05634 (17) Å] is longer than that in (II) [2.966 (1) Å], and the Cl—Au···Au—Cl torsion angle [–65.29 (3)°] is larger than that in (II) [–63.92 (7)°]. The other bond distances and angles in (I) are similar to those in (II).

The crystal structure of (I) is stabilized by several intermolecular C—H···π interactions. Each complex molecule is connected with four adjacent molecules through a C—H···π interaction [H15···*Cg*^i^ = 2.82 Å and H21···*Cg*^ii ^= 2.84 Å; symmetry codes: (i) *x*, 3/2–y, -1/2 + *z*, (ii) 1 - *x*, -1/2 + *y*, 1/2–z]; *Cg* is the centroid of the C25–C30 ring] to construct a two-dimensional sheet structure (Fig. 2). Such an intermolecular C—H···π interaction has not been observed in (II).

### Experimental

To a solution containing tetrahydrothiophenechlorogold(I) (100 mg, 0.32 mmol) in 10 ml of CH_2_Cl_2_ was added *o-*phenylenebis(diphenylphosphine) (140 mg, 0.31 mmol). After stirring for 20 minutes, 100 ml of diethylether was added to the reaction solution. The resulting white powder was recrystallized from CH_2_Cl_2_ by diffusing diethylether, which afforded colorless block crystals of (I).

### Refinement

H atoms were placed at calculated positions and refined with isotropic displacement parameters [*U*_iso_(H) = 1.2*U*_eq_(C)] and a riding model (C—H = 0.95 Å).

### Crystal data

**Table:**

[Au_2_Cl_2_(C_30_H_24_P_2_)]	*F*(000) = 1704
*M**_r_* = 911.27	*D*_x_ = 2.150 Mg m^−^^3^
Monoclinic, *P*2_1_/*c*	Mo *K*α radiation, λ = 0.71075 Å
Hall symbol: -P 2ybc	Cell parameters from 24394 reflections
*a* = 13.0733 (2) Å	θ = 3.1–27.5°
*b* = 12.4206 (2) Å	µ = 10.73 mm^−^^1^
*c* = 17.4630 (3) Å	*T* = 200 K
β = 96.795 (7)°	Block, white
*V* = 2815.69 (8) Å^3^	0.15 × 0.10 × 0.10 mm
*Z* = 4	

### Data collection

**Table:**

Rigaku R-AXIS VII diffractometer	6438 independent reflections
Radiation source: fine-focus sealed tube	5897 reflections with *I* > 2σ(*I*)
graphite	*R*_int_ = 0.030
Detector resolution: 10.000 pixels mm^-1^	θ_max_ = 27.5°, θ_min_ = 3.1°
ω scans	*h* = −16→16
Absorption correction: multi-scan (*ABSCOR*; Higashi, 1995)	*k* = −15→16
*T*_min_ = 0.189, *T*_max_ = 0.341	*l* = −22→22
31710 measured reflections	

### Refinement

**Table:**

Refinement on *F*^2^	Primary atom site location: structure-invariant direct methods
Least-squares matrix: full	Secondary atom site location: difference Fourier map
*R*[*F*^2^ > 2σ(*F*^2^)] = 0.023	Hydrogen site location: inferred from neighbouring sites
*wR*(*F*^2^) = 0.041	H-atom parameters constrained
*S* = 1.16	*w* = 1/[σ^2^(*F*_o_^2^) + (0.0112*P*)^2^ + 2.7453*P*] where *P* = (*F*_o_^2^ + 2*F*_c_^2^)/3
6438 reflections	(Δ/σ)_max_ = 0.002
325 parameters	Δρ_max_ = 0.54 e Å^−^^3^
0 restraints	Δρ_min_ = −0.80 e Å^−^^3^

### Special details

**Table:**

Geometry. All e.s.d.'s (except the e.s.d. in the dihedral angle between two l.s. planes) are estimated using the full covariance matrix. The cell e.s.d.'s are taken into account individually in the estimation of e.s.d.'s in distances, angles and torsion angles; correlations between e.s.d.'s in cell parameters are only used when they are defined by crystal symmetry. An approximate (isotropic) treatment of cell e.s.d.'s is used for estimating e.s.d.'s involving l.s. planes.
Refinement. Refinement of *F*^2^ against ALL reflections. The weighted *R*-factor *wR* and goodness of fit *S* are based on *F*^2^, conventional *R*-factors *R* are based on *F*, with *F* set to zero for negative *F*^2^. The threshold expression of *F*^2^ > σ(*F*^2^) is used only for calculating *R*-factors(gt) *etc*. and is not relevant to the choice of reflections for refinement. *R*-factors based on *F*^2^ are statistically about twice as large as those based on *F*, and *R*- factors based on ALL data will be even larger.

### Fractional atomic coordinates and isotropic or equivalent isotropic displacement parameters (Å^2^)

**Table:**

	*x*	*y*	*z*	*U*_iso_*/*U*_eq_	
Au1	0.283249 (9)	0.406709 (9)	0.296038 (6)	0.02169 (4)	
Au2	0.229077 (9)	0.602225 (9)	0.389584 (6)	0.02065 (4)	
Cl1	0.38861 (7)	0.31036 (7)	0.38364 (5)	0.0385 (2)	
Cl2	0.13663 (7)	0.53399 (7)	0.48118 (5)	0.0371 (2)	
P1	0.18427 (6)	0.49382 (6)	0.20367 (4)	0.01918 (16)	
P2	0.33234 (6)	0.68257 (6)	0.31436 (4)	0.01836 (16)	
C1	0.0679 (2)	0.5541 (2)	0.23080 (17)	0.0234 (7)	
C2	0.0205 (3)	0.5115 (3)	0.29040 (19)	0.0340 (8)	
H2	0.0486	0.4490	0.3164	0.041*	
C3	−0.0664 (3)	0.5576 (3)	0.3129 (2)	0.0444 (10)	
H3	−0.0972	0.5282	0.3548	0.053*	
C4	−0.1088 (3)	0.6462 (3)	0.2748 (2)	0.0444 (10)	
H4	−0.1690	0.6783	0.2904	0.053*	
C5	−0.0643 (3)	0.6886 (3)	0.2142 (3)	0.0523 (11)	
H5	−0.0945	0.7492	0.1872	0.063*	
C6	0.0241 (3)	0.6436 (3)	0.1923 (2)	0.0379 (9)	
H6	0.0550	0.6738	0.1507	0.045*	
C7	0.1435 (2)	0.4040 (2)	0.12379 (17)	0.0236 (7)	
C8	0.0401 (3)	0.3877 (3)	0.0987 (2)	0.0380 (9)	
H8	−0.0107	0.4299	0.1194	0.046*	
C9	0.0104 (3)	0.3101 (3)	0.0436 (2)	0.0502 (10)	
H9	−0.0606	0.2972	0.0279	0.060*	
C10	0.0833 (3)	0.2523 (3)	0.0118 (2)	0.0463 (10)	
H10	0.0626	0.2002	−0.0267	0.056*	
C11	0.1864 (3)	0.2685 (3)	0.03467 (19)	0.0402 (9)	
H11	0.2365	0.2285	0.0115	0.048*	
C12	0.2168 (3)	0.3431 (3)	0.09162 (19)	0.0328 (8)	
H12	0.2879	0.3527	0.1088	0.039*	
C13	0.2511 (2)	0.6058 (2)	0.16336 (17)	0.0197 (6)	
C14	0.2447 (3)	0.6146 (2)	0.08372 (18)	0.0280 (7)	
H14	0.2084	0.5613	0.0523	0.034*	
C15	0.2902 (3)	0.6993 (3)	0.04926 (17)	0.0306 (7)	
H15	0.2860	0.7031	−0.0053	0.037*	
C16	0.3408 (3)	0.7769 (3)	0.09325 (17)	0.0311 (8)	
H16	0.3711	0.8358	0.0695	0.037*	
C17	0.3483 (2)	0.7703 (2)	0.17310 (17)	0.0269 (7)	
H17	0.3840	0.8250	0.2035	0.032*	
C18	0.3046 (2)	0.6855 (2)	0.20917 (16)	0.0202 (6)	
C19	0.4619 (2)	0.6301 (2)	0.33296 (16)	0.0209 (6)	
C20	0.5251 (3)	0.6146 (3)	0.27570 (18)	0.0305 (7)	
H20	0.5006	0.6304	0.2235	0.037*	
C21	0.6239 (3)	0.5762 (3)	0.2947 (2)	0.0399 (9)	
H21	0.6671	0.5651	0.2553	0.048*	
C22	0.6606 (3)	0.5538 (3)	0.37001 (19)	0.0325 (8)	
H22	0.7287	0.5276	0.3826	0.039*	
C23	0.5983 (3)	0.5693 (3)	0.42694 (19)	0.0298 (7)	
H23	0.6237	0.5544	0.4791	0.036*	
C24	0.4994 (2)	0.6062 (2)	0.40878 (18)	0.0271 (7)	
H24	0.4563	0.6156	0.4484	0.032*	
C25	0.3414 (2)	0.8233 (2)	0.34357 (16)	0.0216 (6)	
C26	0.2503 (3)	0.8795 (3)	0.34737 (19)	0.0321 (8)	
H26	0.1862	0.8474	0.3287	0.039*	
C27	0.2528 (3)	0.9822 (3)	0.3782 (2)	0.0422 (9)	
H27	0.1904	1.0207	0.3805	0.051*	
C28	0.3447 (4)	1.0283 (3)	0.4055 (2)	0.0490 (11)	
H28	0.3458	1.0983	0.4275	0.059*	
C29	0.4353 (4)	0.9744 (3)	0.4013 (2)	0.0462 (10)	
H29	0.4990	1.0076	0.4195	0.055*	
C30	0.4338 (3)	0.8710 (3)	0.37043 (19)	0.0339 (8)	
H30	0.4965	0.8334	0.3679	0.041*	

### Atomic displacement parameters (Å^2^)

**Table:**

	*U*^11^	*U*^22^	*U*^33^	*U*^12^	*U*^13^	*U*^23^
Au1	0.02405 (7)	0.02000 (6)	0.02113 (6)	−0.00025 (5)	0.00320 (5)	0.00152 (4)
Au2	0.02083 (7)	0.02433 (7)	0.01768 (6)	−0.00245 (5)	0.00596 (5)	0.00182 (4)
Cl1	0.0439 (5)	0.0344 (5)	0.0352 (5)	0.0106 (4)	−0.0034 (4)	0.0077 (4)
Cl2	0.0305 (4)	0.0520 (5)	0.0309 (4)	−0.0058 (4)	0.0116 (4)	0.0160 (4)
P1	0.0189 (4)	0.0204 (4)	0.0184 (4)	−0.0017 (3)	0.0030 (3)	−0.0002 (3)
P2	0.0203 (4)	0.0216 (4)	0.0138 (4)	−0.0025 (3)	0.0045 (3)	−0.0004 (3)
C1	0.0204 (16)	0.0254 (16)	0.0246 (16)	−0.0056 (13)	0.0039 (13)	−0.0075 (12)
C2	0.0252 (18)	0.046 (2)	0.0313 (19)	−0.0061 (16)	0.0074 (15)	0.0009 (15)
C3	0.030 (2)	0.069 (3)	0.038 (2)	−0.0064 (19)	0.0160 (17)	−0.0050 (19)
C4	0.028 (2)	0.048 (2)	0.061 (3)	−0.0017 (18)	0.0181 (19)	−0.025 (2)
C5	0.041 (2)	0.029 (2)	0.090 (3)	0.0116 (18)	0.022 (2)	−0.004 (2)
C6	0.036 (2)	0.0316 (19)	0.048 (2)	0.0066 (16)	0.0167 (18)	0.0031 (16)
C7	0.0255 (17)	0.0236 (16)	0.0214 (16)	−0.0004 (13)	0.0015 (13)	0.0007 (12)
C8	0.0298 (19)	0.037 (2)	0.046 (2)	0.0058 (16)	−0.0034 (17)	−0.0149 (16)
C9	0.041 (2)	0.052 (2)	0.053 (3)	−0.003 (2)	−0.0140 (19)	−0.019 (2)
C10	0.067 (3)	0.034 (2)	0.035 (2)	0.0033 (19)	−0.010 (2)	−0.0139 (16)
C11	0.058 (3)	0.0335 (19)	0.0293 (19)	0.0136 (18)	0.0077 (18)	−0.0084 (15)
C12	0.032 (2)	0.0336 (18)	0.0331 (19)	0.0030 (15)	0.0070 (15)	−0.0048 (14)
C13	0.0181 (15)	0.0222 (15)	0.0190 (15)	0.0019 (12)	0.0030 (12)	0.0018 (11)
C14	0.0326 (18)	0.0284 (17)	0.0222 (16)	−0.0012 (14)	0.0000 (14)	0.0000 (13)
C15	0.042 (2)	0.0367 (18)	0.0139 (15)	−0.0024 (16)	0.0064 (14)	0.0026 (13)
C16	0.041 (2)	0.0308 (18)	0.0217 (16)	−0.0092 (15)	0.0065 (15)	0.0051 (13)
C17	0.0331 (19)	0.0278 (17)	0.0198 (15)	−0.0072 (14)	0.0028 (14)	−0.0021 (12)
C18	0.0191 (15)	0.0271 (16)	0.0148 (14)	−0.0009 (12)	0.0032 (12)	0.0011 (11)
C19	0.0217 (16)	0.0222 (15)	0.0194 (15)	−0.0022 (12)	0.0050 (12)	−0.0005 (11)
C20	0.0272 (18)	0.046 (2)	0.0187 (16)	0.0034 (15)	0.0047 (14)	−0.0029 (14)
C21	0.0284 (19)	0.065 (2)	0.0280 (19)	0.0073 (18)	0.0101 (16)	−0.0085 (17)
C22	0.0213 (17)	0.0395 (19)	0.036 (2)	0.0058 (15)	0.0017 (15)	−0.0024 (15)
C23	0.0304 (19)	0.0355 (18)	0.0228 (17)	0.0012 (15)	0.0004 (14)	0.0043 (13)
C24	0.0256 (17)	0.0354 (18)	0.0212 (16)	0.0027 (14)	0.0072 (13)	0.0015 (13)
C25	0.0299 (17)	0.0228 (15)	0.0131 (14)	−0.0030 (13)	0.0068 (13)	0.0013 (11)
C26	0.043 (2)	0.0284 (17)	0.0262 (17)	0.0031 (16)	0.0101 (16)	0.0053 (13)
C27	0.066 (3)	0.0287 (19)	0.035 (2)	0.0113 (19)	0.0192 (19)	0.0077 (15)
C28	0.097 (4)	0.0248 (18)	0.0281 (19)	−0.001 (2)	0.019 (2)	−0.0004 (15)
C29	0.074 (3)	0.033 (2)	0.030 (2)	−0.021 (2)	0.0001 (19)	−0.0035 (15)
C30	0.041 (2)	0.0309 (18)	0.0297 (18)	−0.0079 (16)	0.0021 (16)	−0.0017 (14)

### Geometric parameters (Å, °)

**Table:**

Au1—P1	2.2256 (8)	C13—C14	1.388 (4)
Au1—Cl1	2.2739 (8)	C13—C18	1.407 (4)
Au1—Au2	3.05634 (17)	C14—C15	1.381 (4)
Au2—P2	2.2279 (7)	C14—H14	0.9500
Au2—Cl2	2.2792 (8)	C15—C16	1.355 (4)
P1—C1	1.808 (3)	C15—H15	0.9500
P1—C7	1.816 (3)	C16—C17	1.389 (4)
P1—C13	1.827 (3)	C16—H16	0.9500
P2—C19	1.809 (3)	C17—C18	1.384 (4)
P2—C25	1.821 (3)	C17—H17	0.9500
P2—C18	1.830 (3)	C19—C20	1.384 (4)
C1—C2	1.378 (4)	C19—C24	1.388 (4)
C1—C6	1.387 (5)	C20—C21	1.381 (5)
C2—C3	1.372 (5)	C20—H20	0.9500
C2—H2	0.9500	C21—C22	1.373 (5)
C3—C4	1.368 (6)	C21—H21	0.9500
C3—H3	0.9500	C22—C23	1.371 (5)
C4—C5	1.373 (6)	C22—H22	0.9500
C4—H4	0.9500	C23—C24	1.373 (5)
C5—C6	1.377 (5)	C23—H23	0.9500
C5—H5	0.9500	C24—H24	0.9500
C6—H6	0.9500	C25—C30	1.377 (4)
C7—C8	1.385 (5)	C25—C26	1.388 (4)
C7—C12	1.391 (4)	C26—C27	1.383 (5)
C8—C9	1.385 (5)	C26—H26	0.9500
C8—H8	0.9500	C27—C28	1.364 (6)
C9—C10	1.362 (5)	C27—H27	0.9500
C9—H9	0.9500	C28—C29	1.370 (6)
C10—C11	1.374 (5)	C28—H28	0.9500
C10—H10	0.9500	C29—C30	1.392 (5)
C11—C12	1.383 (5)	C29—H29	0.9500
C11—H11	0.9500	C30—H30	0.9500
C12—H12	0.9500		
			
P1—Au1—Cl1	175.87 (3)	C14—C13—C18	118.8 (3)
P1—Au1—Au2	81.343 (19)	C14—C13—P1	118.1 (2)
Cl1—Au1—Au2	102.64 (2)	C18—C13—P1	123.1 (2)
P2—Au2—Cl2	171.48 (3)	C15—C14—C13	121.3 (3)
P2—Au2—Au1	81.132 (19)	C15—C14—H14	119.4
Cl2—Au2—Au1	104.79 (2)	C13—C14—H14	119.4
C1—P1—C7	106.00 (14)	C16—C15—C14	120.1 (3)
C1—P1—C13	103.96 (14)	C16—C15—H15	120.0
C7—P1—C13	106.38 (14)	C14—C15—H15	120.0
C1—P1—Au1	116.51 (10)	C15—C16—C17	119.9 (3)
C7—P1—Au1	110.53 (10)	C15—C16—H16	120.0
C13—P1—Au1	112.70 (10)	C17—C16—H16	120.0
C19—P2—C25	105.53 (14)	C18—C17—C16	121.2 (3)
C19—P2—C18	104.91 (13)	C18—C17—H17	119.4
C25—P2—C18	105.09 (13)	C16—C17—H17	119.4
C19—P2—Au2	110.65 (10)	C17—C18—C13	118.8 (3)
C25—P2—Au2	106.77 (9)	C17—C18—P2	115.4 (2)
C18—P2—Au2	122.62 (10)	C13—C18—P2	125.6 (2)
C2—C1—C6	118.5 (3)	C20—C19—C24	119.1 (3)
C2—C1—P1	120.4 (3)	C20—C19—P2	123.1 (2)
C6—C1—P1	121.1 (2)	C24—C19—P2	117.7 (2)
C3—C2—C1	121.1 (3)	C21—C20—C19	119.7 (3)
C3—C2—H2	119.4	C21—C20—H20	120.1
C1—C2—H2	119.4	C19—C20—H20	120.1
C4—C3—C2	119.9 (3)	C22—C21—C20	120.7 (3)
C4—C3—H3	120.0	C22—C21—H21	119.6
C2—C3—H3	120.0	C20—C21—H21	119.6
C3—C4—C5	119.9 (3)	C23—C22—C21	119.7 (3)
C3—C4—H4	120.0	C23—C22—H22	120.1
C5—C4—H4	120.0	C21—C22—H22	120.1
C4—C5—C6	120.3 (4)	C22—C23—C24	120.2 (3)
C4—C5—H5	119.8	C22—C23—H23	119.9
C6—C5—H5	119.8	C24—C23—H23	119.9
C5—C6—C1	120.1 (3)	C23—C24—C19	120.5 (3)
C5—C6—H6	119.9	C23—C24—H24	119.7
C1—C6—H6	119.9	C19—C24—H24	119.7
C8—C7—C12	119.1 (3)	C30—C25—C26	119.3 (3)
C8—C7—P1	121.3 (2)	C30—C25—P2	122.2 (2)
C12—C7—P1	119.4 (3)	C26—C25—P2	117.9 (2)
C9—C8—C7	120.3 (3)	C27—C26—C25	120.1 (3)
C9—C8—H8	119.9	C27—C26—H26	119.9
C7—C8—H8	119.9	C25—C26—H26	119.9
C10—C9—C8	119.8 (4)	C28—C27—C26	120.1 (4)
C10—C9—H9	120.1	C28—C27—H27	120.0
C8—C9—H9	120.1	C26—C27—H27	120.0
C9—C10—C11	120.9 (3)	C27—C28—C29	120.5 (3)
C9—C10—H10	119.5	C27—C28—H28	119.8
C11—C10—H10	119.5	C29—C28—H28	119.7
C10—C11—C12	119.7 (3)	C28—C29—C30	119.9 (4)
C10—C11—H11	120.2	C28—C29—H29	120.0
C12—C11—H11	120.2	C30—C29—H29	120.0
C11—C12—C7	120.1 (3)	C25—C30—C29	120.0 (4)
C11—C12—H12	119.9	C25—C30—H30	120.0
C7—C12—H12	119.9	C29—C30—H30	120.0
			
P1—Au1—Au2—P2	−70.42 (3)	P1—C13—C14—C15	177.7 (3)
Cl1—Au1—Au2—P2	108.45 (3)	C13—C14—C15—C16	−1.1 (5)
P1—Au1—Au2—Cl2	115.85 (3)	C14—C15—C16—C17	1.0 (5)
Cl1—Au1—Au2—Cl2	−65.29 (3)	C15—C16—C17—C18	−0.1 (5)
Au2—Au1—P1—C1	−42.04 (11)	C16—C17—C18—C13	−0.7 (5)
Au2—Au1—P1—C7	−163.09 (11)	C16—C17—C18—P2	174.4 (3)
Au2—Au1—P1—C13	78.02 (10)	C14—C13—C18—C17	0.6 (4)
Au1—Au2—P2—C19	−63.84 (10)	P1—C13—C18—C17	−176.6 (2)
Au1—Au2—P2—C25	−178.20 (11)	C14—C13—C18—P2	−173.9 (2)
Au1—Au2—P2—C18	60.75 (11)	P1—C13—C18—P2	8.9 (4)
C7—P1—C1—C2	96.0 (3)	C19—P2—C18—C17	−78.0 (3)
C13—P1—C1—C2	−152.0 (3)	C25—P2—C18—C17	33.0 (3)
Au1—P1—C1—C2	−27.4 (3)	Au2—P2—C18—C17	154.9 (2)
C7—P1—C1—C6	−83.8 (3)	C19—P2—C18—C13	96.7 (3)
C13—P1—C1—C6	28.2 (3)	C25—P2—C18—C13	−152.3 (3)
Au1—P1—C1—C6	152.8 (2)	Au2—P2—C18—C13	−30.5 (3)
C6—C1—C2—C3	−1.7 (5)	C25—P2—C19—C20	−101.8 (3)
P1—C1—C2—C3	178.5 (3)	C18—P2—C19—C20	9.0 (3)
C1—C2—C3—C4	1.4 (6)	Au2—P2—C19—C20	143.1 (2)
C2—C3—C4—C5	0.1 (6)	C25—P2—C19—C24	77.1 (3)
C3—C4—C5—C6	−1.2 (6)	C18—P2—C19—C24	−172.2 (2)
C4—C5—C6—C1	0.8 (6)	Au2—P2—C19—C24	−38.1 (3)
C2—C1—C6—C5	0.6 (5)	C24—C19—C20—C21	−0.1 (5)
P1—C1—C6—C5	−179.6 (3)	P2—C19—C20—C21	178.7 (3)
C1—P1—C7—C8	−3.9 (3)	C19—C20—C21—C22	−0.5 (6)
C13—P1—C7—C8	−114.2 (3)	C20—C21—C22—C23	0.2 (6)
Au1—P1—C7—C8	123.2 (3)	C21—C22—C23—C24	0.6 (5)
C1—P1—C7—C12	−178.1 (2)	C22—C23—C24—C19	−1.1 (5)
C13—P1—C7—C12	71.6 (3)	C20—C19—C24—C23	0.9 (5)
Au1—P1—C7—C12	−51.0 (3)	P2—C19—C24—C23	−178.0 (2)
C12—C7—C8—C9	1.4 (5)	C19—P2—C25—C30	2.3 (3)
P1—C7—C8—C9	−172.8 (3)	C18—P2—C25—C30	−108.3 (3)
C7—C8—C9—C10	−2.4 (6)	Au2—P2—C25—C30	120.1 (2)
C8—C9—C10—C11	1.2 (6)	C19—P2—C25—C26	−169.8 (2)
C9—C10—C11—C12	1.1 (6)	C18—P2—C25—C26	79.7 (3)
C10—C11—C12—C7	−2.1 (5)	Au2—P2—C25—C26	−52.0 (2)
C8—C7—C12—C11	0.9 (5)	C30—C25—C26—C27	−0.3 (5)
P1—C7—C12—C11	175.2 (3)	P2—C25—C26—C27	172.0 (2)
C1—P1—C13—C14	−101.0 (3)	C25—C26—C27—C28	−0.4 (5)
C7—P1—C13—C14	10.6 (3)	C26—C27—C28—C29	1.2 (5)
Au1—P1—C13—C14	131.9 (2)	C27—C28—C29—C30	−1.2 (5)
C1—P1—C13—C18	76.2 (3)	C26—C25—C30—C29	0.3 (5)
C7—P1—C13—C18	−172.1 (2)	P2—C25—C30—C29	−171.7 (3)
Au1—P1—C13—C18	−50.9 (3)	C28—C29—C30—C25	0.5 (5)
C18—C13—C14—C15	0.3 (5)		

### Hydrogen-bond geometry (Å, °)

**Table:**

*D*—H···*A*	*D*—H	H···*A*	*D*···*A*	*D*—H···*A*
C15—H15···Cg^i^	0.95	2.82	3.569 (4)	137
C21—H21···Cg^ii^	0.95	2.84	3.559 (4)	134

Symmetry codes: (i) *x*, −*y*+3/2, *z*−1/2; (ii) −*x*+1, *y*−1/2, −*z*+1/2.
